# Hydrogenation treatment under several gigapascals assists diffusionless transformation in a face-centered cubic steel

**DOI:** 10.1038/s41598-021-98938-1

**Published:** 2021-09-29

**Authors:** Motomichi Koyama, Hiroyuki Saitoh, Toyoto Sato, Shin-ichi Orimo, Eiji Akiyama

**Affiliations:** 1 Institute for Materials Research, Tohoku University, 2-1-1 Katahira, Aoba-ku, Sendai, Miyagi 980-8577 Japan; 2 Elements Strategy Initiative for Structural Materials, Kyoto University, Yoshida-honmachi, Sakyo-ku, Kyoto, 606-8501 Japan; 3 National Institutes for Quantum and Radiological Science and Technology, 1-1-1 Kouto, Sayo, Hyogo 679-5148 Japan; 4 Department of Engineering Science and Mechanics, Shibaura Institute of Technology, 3-7-5 Toyosu, Koto-ku, Tokyo, 135-8548 Japan; 5 WPI-Advanced Institute for Materials Research, Tohoku University, 2-1-1 Katahira, Aoba-ku, Sendai, Miyagi 980-8577 Japan

**Keywords:** Structural materials, Metals and alloys

## Abstract

The use of hydrogen in iron and steel has the potential to improve mechanical properties via altering the phase stability and dislocation behavior. When hydrogen is introduced under several gigapascals, a stoichiometric composition of hydrogen can be introduced for steel compositions. In this study, a face-centered cubic (fcc) stainless steel was hydrogenated under several gigapascals. When the steel was not hydrogenated, the microstructure after depressurization was an fcc with a hexagonal close-packed (hcp) structure. In contrast, the hydrogenation treatment resulted in a fine lath body-centered cubic (bcc) structure arising from diffusionless transformation. In particular, the bcc phase formed through the following transformation sequence: fcc → hcp → dhcp (double hexagonal close-packed phase) → bcc. That is, the use of hydrogenation treatment realized fine microstructure evolution through a new type of diffusionless transformation sequence, which is expected to be used in future alloy design strategies for developing high-strength steels.

## Introduction

In metals, microstructure control has historically been the key to achieving superior mechanical properties. Specifically, refinement of microstructure and control of lattice defect behavior are intrinsically important for realizing high strength with significant ductility. In this context, the occurrence of diffusionless transformation is the origin of the high performance of steels^1^, which enables microstructure refinement and introduces a considerable amount of dislocations^2,3^. In this study, transformation that does not involve the diffusion of ‘‘substitutional atoms” has been referred to as diffusionless transformation. The conditions required for inducing diffusionless transformation are low diffusivity of the substitutional atoms (or high cooling rate) and the presence of a significant driving force arising from the difference between the free energies of the two arbitrary phases. In the case of steels, the face-centered cubic (fcc) to body-centered cubic (bcc) diffusionless transformation, which has been practically used to increase the strength, generally occurs during the cooling and deformation stages. In terms of chemical composition, the addition of interstitial elements, such as carbon and nitrogen^4,5^, plays a key role in controlling the diffusionless transformation in steels.

The strength of the diffusionless transformation phase increases with increasing interstitial atom content, microstructure boundary density, and dislocation density. Therefore, one of the major targets in the field of steel strength is to use the interstitial elements to control the substructure of the diffusionless transformation phase. Hydrogen is also an interstitial element in steels^6^, and it has recently been noted as a promising element that enables the control of microstructures associated with the diffusionless transformation^7–9^. Moreover, a large amount of hydrogen can be introduced into steel by pressurizing the sample with hydrogen^10,11^. In this study, we present the potential of hydrogen to induce or control diffusionless transformation and demonstrate the dramatic change in the microstructure through hydrogenation in steel.

## Results and discussion

### Microstructure evolution via high-pressure hydrogenation

**Figure 1:**
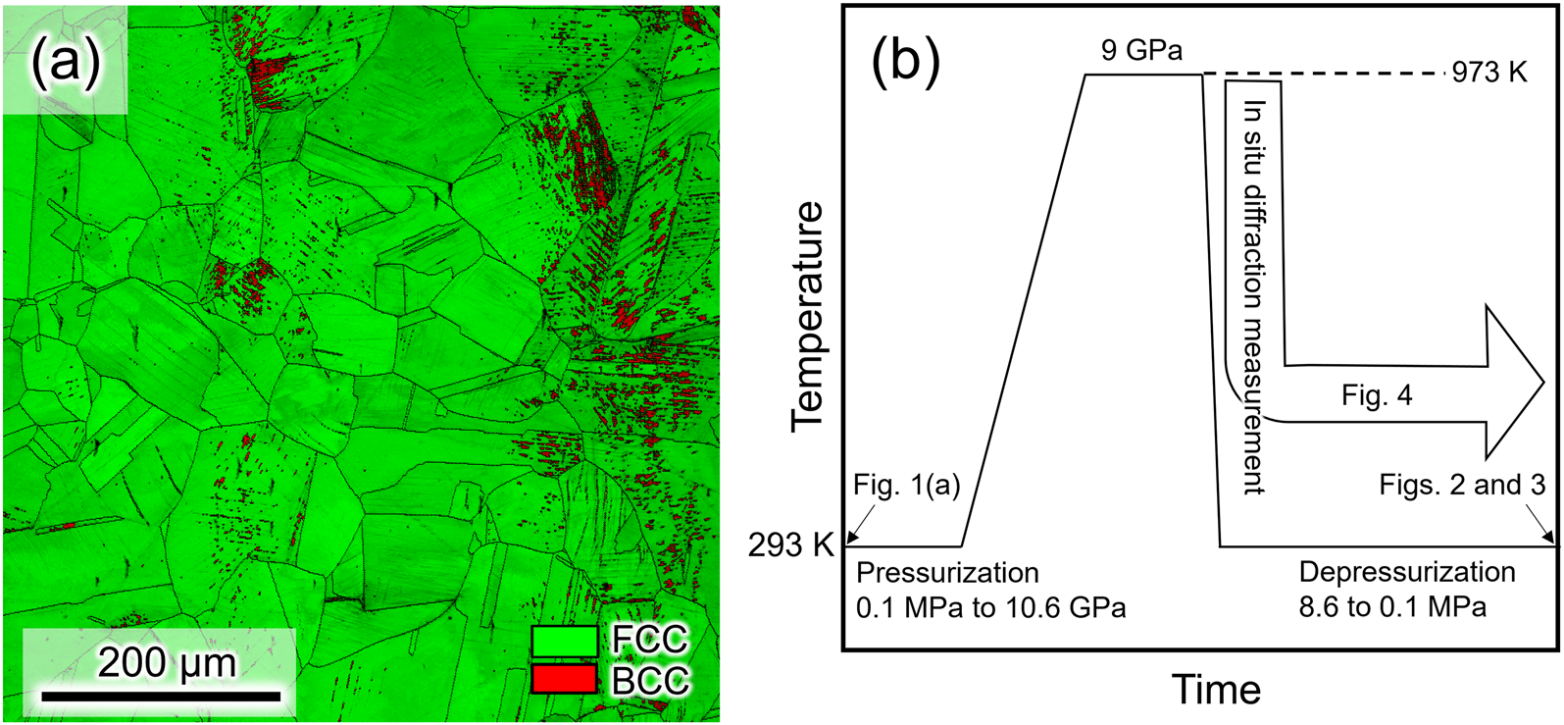
(**a**) Phase map showing the as-solution-treated specimen, and (**b**) schematic for the hydrogenation treatment. The schematic was drawn by power point for Microsoft 365.

**Figure 2:**
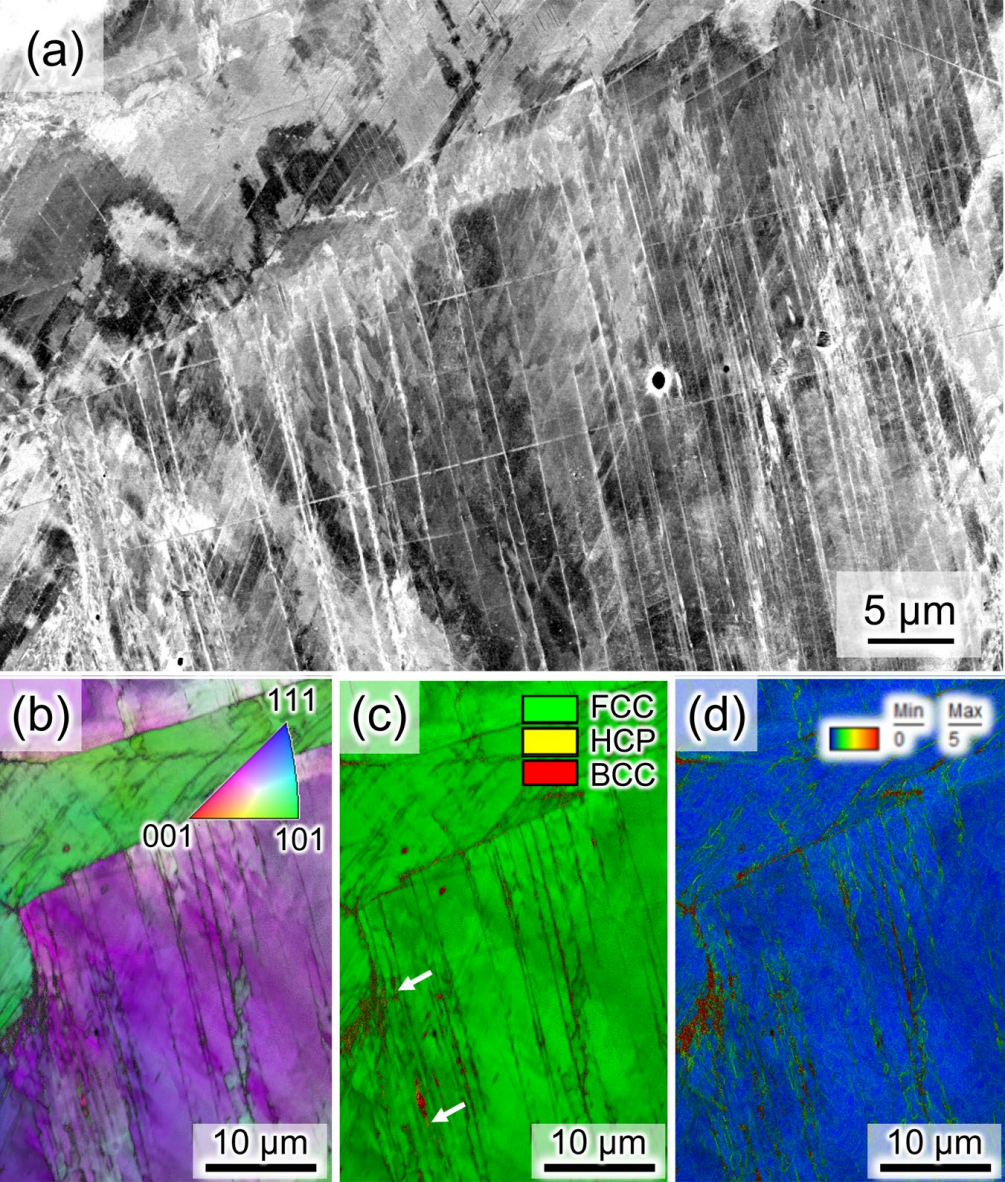
(**a**) Electron channeling contrast (ECC) image showing the microstructure after depressurization to 0.1 MPa in the non-hydrogenated specimen. (**b**) Inverse pole figure (IPF), (**c**) phase, and (d) Kernel average misorientation (KAM) maps taken at the identical location to the ECC image, which were overlapped with the gray scale image quality map. That is, the dark contrast in (**a–c**) indicates low image qualities, and the plate-like low-image-quality region implies the presence of hcp phase. The IPF, phase and KAM maps are overlapped with the image quality map. The KAM values correspond to the geometrically necessary dislocation density.

Figure 1a shows the microstructure of a Fe–Cr–Ni-based fcc steel, called type 304L, in the as-solution-treated condition. The microstructure consisted of an fcc phase along with a small amount of a bcc phase. The bcc phase is known to form without diffusion of substitutional atoms. The sample, with the initial microstructure, was pressurized to 10.6 GPa at 293 K. The pressurized specimen showed the microstructure consisting of fcc and hcp phases as shown in Fig. S1. The pressurized specimens were heated to 973 K at 9 GPa where recrystallization and hydrogenation occurred, and subsequently cooled to 293 K and depressurized to 0.1 MPa, as schematically shown in Fig. 1b. After depressurization at 293 K, the microstructure without hydrogenation remained in the fcc phase and contained plate-like products, as shown in Fig. 2a and b. The thin plate-like products, which appear as white contrast in Fig. 2a, are known to be diffusionless transformation phases consisting of the hexagonal close-packed (hcp) structure^12^, which was also confirmed by the in situ XRD measurements shown in the next section. In addition, thick regions of hcp plates were detected in the phase map as indicated by the black arrows in Fig. 2c. A local stress that evolved during the fcc → hcp diffusionless transformation was accommodated by a partial transformation from hcp to bcc or dislocation slip near the hcp plates. Correspondingly, a small amount of the bcc phase and a relatively high dislocation density were observed near or within the hcp plates, as shown in Fig. 2c and d.

**Figure 3:**
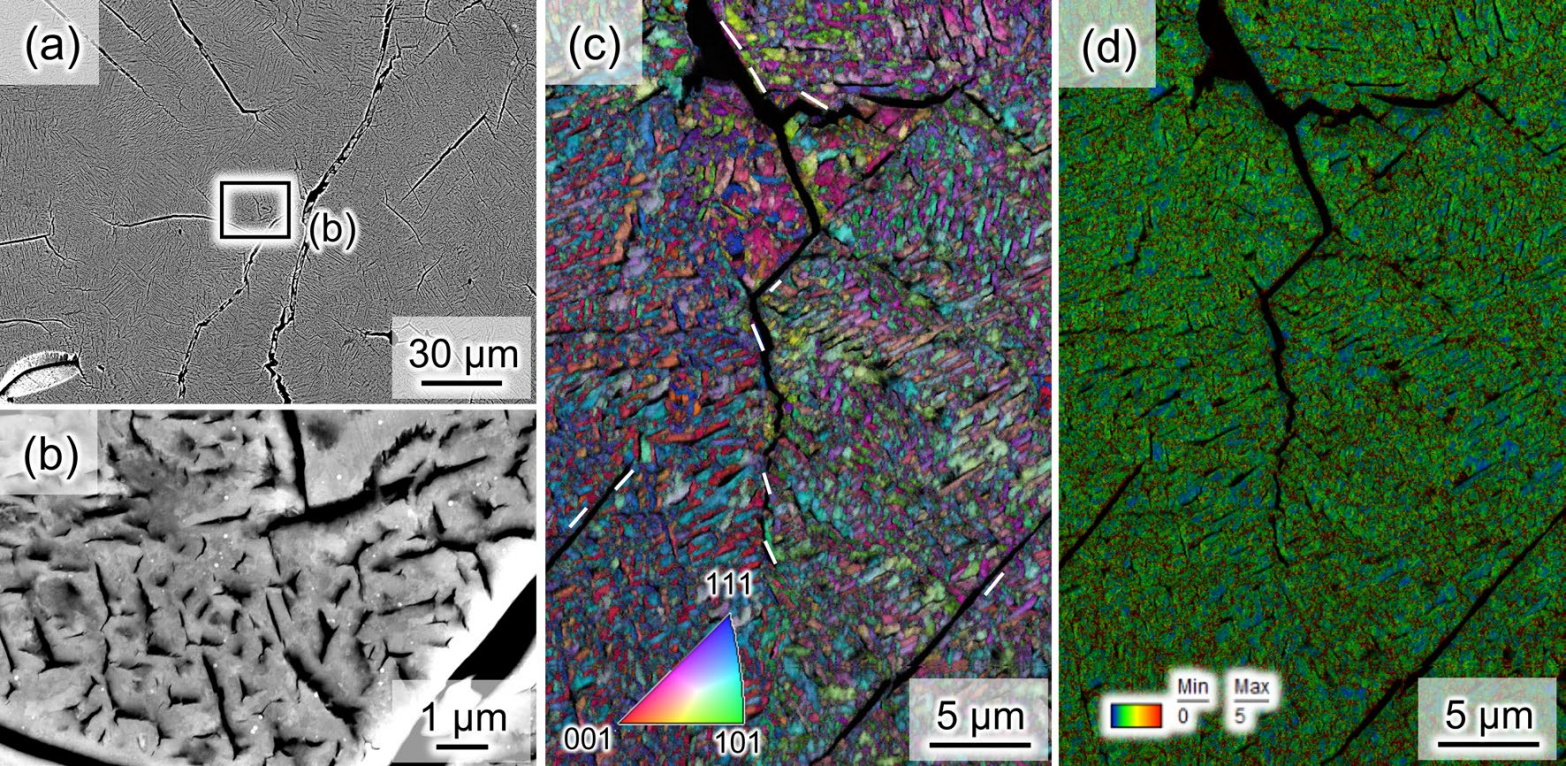
(**a**,**b**) Secondary electron images, (**c**) IPF map (bcc phase), and (**d**) KAM map that show the microstructure and numerous cracks in the hydrogenated specimen after depressurization. The white lines in (**c**) indicate some examples of {110}_bcc_ traces along the cracks. The IPF and KAM maps are overlapped with the image quality map.

An experiment with a similar procedure, but with hydrogenation, was conducted for another sample. Specifically, the sample was pressurized to 9 GPa with a hydrogen source (AlH_3_)^13,14^, heated to 973 K, and subsequently depressurized at 293 K. There were three interesting differences between the samples with and without hydrogenation. The first difference was the formation of numerous tiny cracks (Fig. 3a,b). The second difference was the constituent phase; the hydrogenated microstructure was fully indexed as the bcc phase after depressurization (Fig. 3c). The microstructure morphology was a fine lath, which is a typical bcc diffusionless transformation phase in steels^15^. The third difference was the high dislocation density in the hydrogenated sample compared with dislocation density in the non-hydrogenated sample (Fig. 3d). As the dislocations that were introduced during the pressurization must have been removed during recrystallization at 973 K, a large number of dislocations, observed in Fig. 3d, were introduced during the cooling and depressurization. Most of the cracks were along the {110}_bcc_ plane, which is the typical crystallographic plane of hydrogen-induced cracks in the bcc structure of steels^16,17^. These findings indicate that the hydrogen-related diffusionless transformation to the bcc phase possibly caused the internal stress and the associated introduction of dislocations and cracks. The most important finding here is the fact that “the hydrogenation treatment could refine the microstructure (below 300 nm) and introduce numerous dislocations by promoting diffusionless transformation”. Also, it must be noted that, in general, type 304L steel does not show a microstructure that fully consists of the bcc phase arising from diffusionless transformation as long as the steel is not severely plastically deformed^18,19^. That is, we can say that the hydrogenation treatment could create an unconventional microstructure in fcc steel. In addition, the Vickers hardness values of the specimens with and without hydrogenation were measured to be 308 ± 29 and 261 ± 19 HV, respectively. In order to understand the details of the sequence of the diffusionless bcc transformation, in situ diffraction experiments were carried out and are described in the next section.

### In situ diffraction experiments

**Figure 4:**
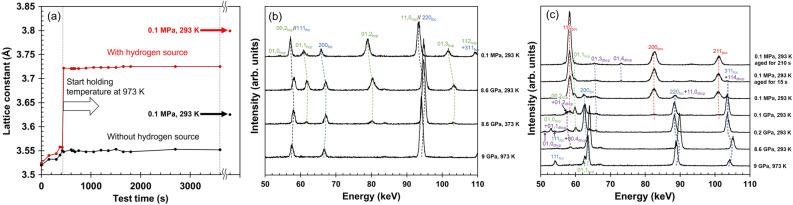
(**a**) Variations of fcc lattice constants obtained by the in situ diffraction experiments and the corresponding profiles taken in the (**b**) non-hydrogenated and (**c**) hydrogenated specimens.

To estimate hydrogen content, the lattice constants obtained by the in situ diffraction experiments are first noted as shown in Fig. 4a. The drastic increase in the lattice constant of the specimen pressurized with the hydrogen source indicates occurrence of the hydrogenation at 973 K and 9GPa. Hydrogen content in the FeH_x_ hydride was estimated to x = 0.76, based on the linear relationship between lattice expansion and hydrogen content^6,20,21^. Figure 4b shows the variations in the diffraction profiles during cooling under high pressure and depressurization in the sample without hydrogenation. The profiles were obtained by in situ synchrotron radiation X-ray diffraction measurements. When the sample was heated to 973 K at 9 GPa, the constituent phase was fully fcc. During the cooling from 973 to 293 K, fcc → hcp transformation occurred. The fcc → hcp transformation proceeded during depressurization to 0.1 MPa. As shown in Fig. 4c, the hydrogenated specimen also indicated that the constituent phase at 973 K was almost a single fcc structure (a small amount of hcp phase might have already formed at this state). Subsequent cooling to 293 K and depressurization to 0.2 GPa resulted in hcp and double hexagonal close-packed (dhcp) phases. Further depressurization to 0.1 GPa caused bcc phase transformation. Depressurization to 0.1 MPa and subsequent aging at 293 K increased the 110_bcc_ peak intensity and decreased the 200_fcc_ and 01,1_hcp_ peak intensities. The 01,0_dhcp_ peak disappeared during depressurization, and the 01,4_dhcp_ peak appeared during aging.

According to the profile variation, the fcc → hcp transformation preferentially occurred, and the dhcp phase formed as a minor phase. Subsequently, bcc transformation occurred, which involved the disappearance of the hcp phase. In addition, the increase in bcc peak intensity and the decrease in hcp peak intensity continued during aging, even after the fcc phase completely disappeared. Moreover, as clarified in the previous section, the bcc transformation in the hydrogenated specimen was of the diffusionless type, and the diffusion of substitutional solute atoms cannot occur at ambient temperature. These observations indicate that the diffusionless transformation from fcc to hcp to bcc occurred during the cooling, depressurization, and aging processes.

In the aspect of dhcp phase, the following facts are noteworthy:

1. The 01,0_dhcp_ peak disappeared during depressurization, and the 01,4_dhcp_ peak appeared during aging after depressurization. These findings indicate that the {01,0}_dhcp_-oriented grains transformed to hcp or bcc phases, and a new dhcp phase appeared later in the {01,4}_dhcp_-oriented grains. It should be noted that the aging effect in hydrogenated iron stems from the partial hydrogen desorption from the matrix^22^. Since the dhcp hydride phase cannot be stabilized by dehydrogenation, these findings indicate that the dhcp phase is an intermediate phase.
2. The 01,4_dhcp_ peak intensity increased during aging, even after fcc disappeared. As bcc is the most stable phase in type 304L when hydrogen is desorbed, the bcc → dhcp transformation is ruled out. That is, hcp → dhcp transformation occurred, which is consistent with a previous study that demonstrated that the hcp phase existed as a metastable intermediate phase in the Fe-H system^21^.
3. When the microstructure was observed by electron backscatter diffraction (EBSD) analyses, it was revealed that the microstructure was fully indexed to the bcc phase. This indicates that the bcc transformation occurred during further aging, which involved the disappearance of the dhcp phase. That is, dhcp → bcc transformation occurred.

These three points indicate that fcc to hcp to dhcp to bcc diffusionless transformation occurred. As discussed in a previous work^22^, the complex structure of dhcp requires complicated lattice invariant shears, which results in the introduction of a high density of dislocations.

In terms of the driving force for the diffusionless transformation, the volumetric strain resulting from local hydrogen desorption may be the key factor. The lattice constants of the fcc phases with and without hydrogenation at 0.1 MPa and 293 K were 3.792 and 3.596 Å, respectively. Therefore, when a major portion of hydrogen in a local area was desorbed, a 14.7% shrinkage strain was provided. When the local area was constrained by the surrounding microstructure, the negative hydrostatic stress corresponding to the shrinkage strain acted as the driving force for the subsequent diffusionless transformation^23^. Alternatively, if hydrogen was not desorbed until the formation of the dhcp phase, the dhcp phase became extremely unstable after the hydrogen content decreased. The large difference in free energy between dhcp and bcc in the dehydrogenated condition can also act as a driving force for the dhcp → bcc transformation during hydrogen desorption. Furthermore, the lattice constant of the bcc phase at 0.1 MPa and 293 K was 2.874 Å, which is close to the lattice constant of the bcc diffusionless transformation phase of type 304 stainless steel without hydrogen (e.g., 2.868 Å^24^ and 2.870 Å^25^). This indicates that a major portion of the hydrogen in the bcc phase was desorbed after and/or during the transformation from fcc to bcc phases. Therefore, there must be a significant misfit between the hydrogenated and dehydrogenated bcc regions during the diffusionless transformation, which can cause hydrogen-assisted cracking of the microstructure boundaries, as shown in Fig. 3.

In summary, the high-pressure hydrogenation treatment could create the new type bcc microstructure in the Fe–Cr–Ni-based fcc steel through the transformation sequence of fcc → hcp → dhcp → bcc. The bcc microstructure showed the fine lath morphology and contained a considerable number of dislocations, which showed a higher hardness than that of the microstructure without hydrogenation treatment. A problematic issue was the appearance of numerous cracks in the microstructure. That is, the hydrogenation treatment is expected to be a new route to create high-strength microstructure in metals when the crack formation can be significantly suppressed.

## Methods

Cylindrical specimens of type SUS304L austenitic stainless steel with a diameter of 1 mm and a height of 0.3 mm were used in this study. The detailed chemical composition was Fe–18.6Cr–9.42Ni–1.36Mn–0.54Si–0.014C–0.014N–0.034P–0.003S in wt%. A SUS304L plate was solution-treated at 1327 K for 30 min and subsequently water-quenched. The heat-treated plate was cut by spark machining to produce the cylindrical specimens. The sample cell assembly drawing is shown in Fig. S2. The specimens were pressurized to several gigapascals using a cubic-type multi-anvil press. Hydrogenation of the specimens was achieved using a high-pressure cell with a compacted AlH_3_ disk as an internal hydrogen source. The AlH_3_ disk decomposed into fluid hydrogen and aluminum metal when it was heated above 800 K, and the fluid hydrogen encapsulated in the NaCl capsule reacted with the iron specimens to form FeH_x_. The iron specimens with and without hydrogenation were first pressurized to 10.6 GPa and then heated to 973 K with a holding time of 2 h. Subsequently, the specimens were cooled to room temperature (⁓ 293 K) at a cooling rate of 100 K/min, and thereafter depressurized from 8.6 GPa to ambient pressure over approximately 2 h. The specimen temperature was estimated from the relation between the generated temperature and input electric power, calibrated with a Pt–Pt 13% Rh thermocouple. The pressure value was calculated based on a relationship between applied load and pressure, which was determined by a separate experiment in which a pressure marker made of NaCl was placed^26^. Accuracies of pressure and temperature values were estimated to be ± 0.3 GPa and ± 20 °C, respectively. Vickers hardness was measured using a Shimadzu microVickers hardness tester HMV-G with a load of 98.7 mN and a holding time for 10 s. The load applied for this experiment was the minimum value in the hardness tester. The indentation was conducted in between large cracks but the effects of small cracks probably along block boundaries could not be avoided in this experiment. Therefore, the hardness of the hydrogenated specimens may have been underrated.

Crystal structure changes were observed via in situ synchrotron radiation X-ray diffraction. White X-rays, generated from a bending magnet source, were irradiated on the specimen, and the diffracted X-rays were detected using a solid-state detector in energy dispersive mode. The diffraction angle 2*θ* was fixed at 6°. The diffraction was continuously recorded during the heat treatment and depressurization processes, and each datum contained an average value of 15 s. Hydrogen content *x* for MH_*x*_ (M is a metal atom) under high pressure is proportional to the volume expansion Δ*v* = *v* (hydride) −* v* (reference metal). Thus, hydrogen content *x* can be calculated using the relation, *x* = Δ*v*/*v*_*H*_*,* where *v*_*H*_ is the hydrogen induced volume. The hydrogen induced volumes for fcc and hcp FeH_*x*_ are 2.21 and 2.48 Å^3^, respectively^6,20,21^. The microstructure and dislocation distribution of the specimens after pressurization, heating, cooling, and depressurization were characterized via electron backscatter diffraction (EBSD) analysis. The EBSD specimens were mechanically polished with colloidal silica, which had a particle size of 60 nm. The EBSD measurements were conducted at 20 kV with a beam step size of 100 nm.

## Supplementary Information

Supplementary Figures.
