# Supplementary material for: Hydrogenation treatment under several gigapascals assists diffusionless transformation in a face-centered cubic steel
Source: Sci Rep. 2021 Sep 29;11:19384. doi: 10.1038/s41598-021-98938-1 (PMC8481468; doi:10.1038/s41598-021-98938-1)
Supplement: Supplementary file 1 — Supplementary Figures. [file 41598_2021_98938_MOESM1_ESM.docx]

**Supplementary information for**

**Hydrogenation treatment under several gigapascals assists diffusionless transformation in a face-centered cubic steel**

Motomichi Koyama^a,b*^, Hiroyuki Saitoh^c^, Toyoto Sato^d^, Shin-ichi Orimo^a,e^, Eiji Akiyama^a^

^a^Institute for Materials Research, Tohoku University, 2-1-1 Katahira, Aoba-ku, Sendai, Miyagi 980-8577, Japan

^b^Elements Strategy Initiative for Structural Materials, Kyoto University, Yoshida-honmachi, Sakyo-ku, Kyoto 606-8501, Japan

^c^National Institutes for Quantum and Radiological Science and Technology, 1-1-1 Kouto, Sayo, Hyogo 679-5148, Japan

^d^Department of Engineering Science and Mechanics, Shibaura Institute of Technology, 3-7-5 Toyosu, Koto-ku, Tokyo 135-8548, Japan

^e^WPI-Advanced Institute for Materials Research, Tohoku University, 2-1-1 Katahira, Aoba-ku, Sendai, Miyagi 980-8577, Japan

*Corresponding author, e-mail: [koyama@imr.tohoku.ac.jp](mailto:koyama@imr.tohoku.ac.jp)


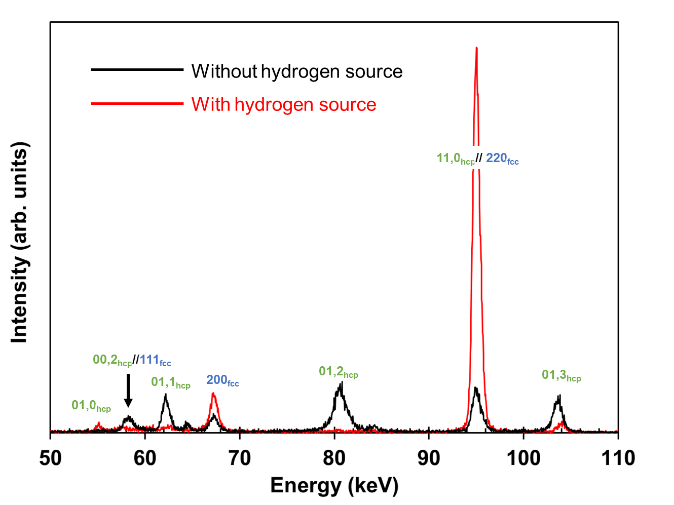


Fig. S1 X-ray diffraction profiles of the specimens immediately after the pressurization to 10.6 GPa at 293 K.


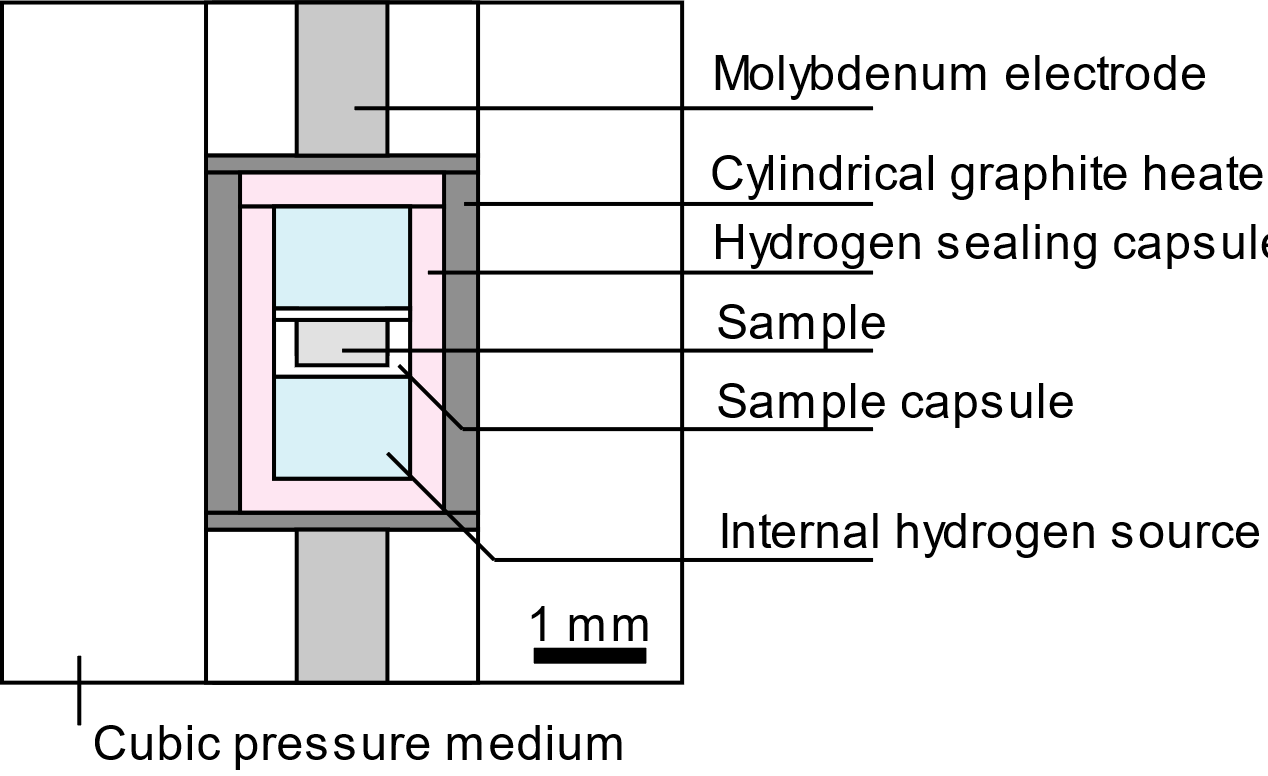


Fig. S2. Schematic illustration of a high-pressure cell assembly. The schematic was drawn by Adobe illustrator 2021.
